# Cognitive Bias Modification (CBM) of obsessive compulsive beliefs

**DOI:** 10.1186/1471-244X-13-256

**Published:** 2013-10-09

**Authors:** Alishia D Williams, Jessica R Grisham

**Affiliations:** 1 The Clinical Research Unit for Anxiety and Depression, School of Psychiatry, The University of New South Wales, Level 4 O’Brien Building at St. Vincent’s Hospital, Sydney, Australia; 2 School of Psychology, The University of New South Wales, Sydney, Australia

**Keywords:** Cognitive bias modification, Interpretation bias, Obsessive compulsive disorder, Maladaptive beliefs

## Abstract

**Background:**

Cognitive bias modification (CBM) protocols have been developed to help establish the causal role of biased cognitive processing in maintaining psychopathology and have demonstrated therapeutic benefits in a range of disorders. The current study evaluated a cognitive bias modification training paradigm designed to target interpretation biases (CBM-I) associated with obsessive compulsive disorder (OCD).

**Methods:**

We evaluated the impact of CBM-I on measures of interpretation bias, distress, and on responses to three OC stressor tasks designed to tap the core belief domains of Importance of Thoughts/Control, Perfectionism/Intolerance of Uncertainty, and Contamination/Estimation of Threat in a selected sample of community members reporting obsessive compulsive (OC) symptoms (N = 89).

**Results:**

Participants randomly assigned to the Positive condition evidenced a change in interpretation bias towards more positive and less negative OC-relevant interpretations following CBM-I compared to participants assigned to the Control condition. Importantly, a positivity bias was not observed for foil scenarios unrelated to the core OC belief domains. Further, participants in the Positive condition reported less distress and urge to neutralize following an OC stressor task designed to tap Importance of Thoughts/Control. No significant difference emerged on the indices of behavioural response to the OC stressor tasks. Severity of OC symptoms did not moderate the effects of positive CBM-I training.

**Conclusions:**

CBM-I appears effective in selectively targeting OC beliefs. Results need to be replicated in clinical samples in order for potential therapeutic benefit to be demonstrated.

## Background

Obsessive compulsive disorder (OCD) is a disorder characterised by intrusive, repetitive thoughts (obsessions), which typically lead to engagement in either mental or behavioural compulsions designed to alleviate associated anxiety and distress. Epidemiology studies have estimated that the lifetime prevalence of OCD ranges from 2% to 3.8% [1-3]. The quality of life of persons with OCD is severely affected [4]. Left untreated, OCD has a chronic course [5]. The treatment of choice is cognitive-behavioural therapy (CBT), but with a response rate of only 50% when drop-out rates are considered [6], there is a clear need for treatment innovation.

One promising area of research that may advance OCD treatments focuses on the role of implicit cognition and biases in the development and maintenance of the disorder. Cognitive biases refer to the tendency to preferentially process negative or threatening information, either through increased allocation of attention resources (*attentional bias*) or via rapid assignment of negative or threatening appraisals to ambiguous information (*interpretive bias*) – for an overview see: [7]. Extensive research has established that anxious individuals preferentially allocate their attention towards threat-related information [8] and interpret ambiguous information in a negative manner [9]. However, leading authorities in the field have noted that although prominent cognitive theories of anxiety implicate attentional and interpretive biases in the onset and maintenance of anxiety disorders [10] the causal nature of these biases remains to be established in all disorders [11].

Accordingly, there has been a recent surge in international interest in the application of novel experimental methodologies to establish the causal role of biased cognitive processes in maintaining anxiety disorders. These developments have underscored the need for greater understanding of disorder-specific biases in OCD. Prominent cognitive models of OCD assert that intrusive thoughts are experienced by most people, but develop into obsessions when appraised as posing a threat for which the individual is personally responsible. Thus by its very nature, OCD is an ideal candidate disorder to examine pathological cognitive biases. Whereas several studies have evaluated the presence of *attentional* biases as a form of aberrant information processing in OCD [12-14], other research has focused on *interpretive* biases. An emphasis on interpretive biases is consistent with leading cognitive models of OCD, which propose that it is the interpretation of an unwanted intrusive thought or image that leads to anxiety, distress, and the concomitant behavioural acts that are the defining features of the disorder [15]. Substantial correlational evidence supports the association between negative interpretations of intrusive thoughts and OC symptoms [16,17]. However, identification of the presence of cognitive biases is insufficient to conclude that these biases causally contribute to psychological dysfunction [18].

Experimental paradigms are required to establish the causal role of cognitive biases in symptom expression and maintenance in OCD. One way to test causal hypotheses is to target biased attention and appraisals via cognitive bias modification (CBM) paradigms. CBM is a cognitive experimental methodology that modifies biases via training conditions in which participants are exposed to a series of stimuli designed to manipulate processing relevant to psychopathology. CBM procedures are either designed to modify an interpretive bias (CBM-I) or an attentional bias (CBM-A). Both types of CBM paradigms have demonstrated efficacy in modifying cognitive biases implicated in the anxiety disorders and the resultant change in selective information processing has been shown to impact upon clinically relevant symptoms [18]. For example, CBM techniques have proven to be effective in reducing clinical symptoms and dysfunction across a range of disorders including depression [19-21], generalised anxiety disorder [22], and social anxiety [23,24]. Importantly, research has demonstrated that the induced adaptive processing biases lead to subsequent reductions in emotional reactivity to subsequent stressor tasks [19,25,26] as well as reductions in symptoms [27].

Evidence is emerging for the impact of CBM-I techniques in OCD. Clerkin and Teachman [28] employed a CBM-I paradigm to evaluate whether participants high in OC symptoms could be trained to adopt healthier interpretations. They tested whether this training influenced participants’ later responses to an OC-stressor task (a task designed to elicit distress and urges to engage in a compulsion or neutralisation behaviour). Consistent with expectations, high OC participants in the positive (versus control) training condition endorsed healthier OC-relevant interpretations and beliefs following training, and reported (at trend level) less negative emotion during the subsequent stressor task after controlling for baseline negative affect.

These results offer support for the cognitive model of OCD, which emphasises the importance of appraisals of intrusive thoughts, and provide initial evidence that modifying interpretation biases in a high OC population may have downstream effects on emotional and physiological responses to OC stressors. The inclusion of a behavioural measure in this study was important to provide a robust test of the effects of CBM on emotional consequences that is not confounded by the potential for self-report bias [29]. However, OCD experts have identified six core appraisals implicated in OCD symptom maintenance: 1) Tolerance for Uncertainty (e.g., '*It is essential for me to consider all possible outcomes of a situation’*), 2) Threat Estimation (e.g., '*I often think things around me are unsafe’*), 3) Control of Thoughts (e.g., '*I should be able to rid my mind of unwanted thoughts’*), 4) Importance of Thoughts (e.g., '*Having nasty thoughts means I am a terrible person’*), 5) Responsibility (e.g., '*If my actions could have even a small effect on a potential misfortune, I am responsible for the outcome’*) and 6) Perfectionism (e.g., '*If I don’t do a job perfectly, people won’t respect me’*). Development of OC-stressor tasks to index anxiety corresponding to each of these domains is necessary to provide a comprehensive evaluation of the downstream effect of CBM on behaviour. Inclusion of a stressor task in this type of research is also important given diathesis-stress conceptualisations that propose negative cognitive biases are latent vulnerability factors that only directly influence symptoms once activated by a disorder-relevant stressor [11]. Further, Clerkin and Teachman [28] employed a student sample all high in OC symptoms. In the absence of a low OC symptom or healthy control group, the specificity of the effect to OC symptoms remains unknown. It is possible that this form of CBM training could have generic positive effects irrespective of OC symptom level.

Therefore the aims of the current study were to replicate and extend the findings of Clerkin and Teachman by evaluating a CBM-I training paradigm in a selected sample of community members with varying levels of OC symptoms. We aimed to assess the impact of CBM-I on measures of interpretation bias, distress, and on responses to three behavioural tasks designed to tap the core belief domains of Importance of Thoughts/Control, Perfectionism/Intolerance of Uncertainty, and Contamination/Estimation of Threat.

On the basis of previous finding, we predicted that participants assigned to the Positive CBM-I training condition would evidence a change in interpretation bias towards more positive and less negative OC-relevant interpretations compared to participants assigned to the Control CBM-I training condition. Further, we predicted that participants in the Positive CBM-I training condition would report less distress and evidence a more adaptive response (defined in relation to each task) to the behavioural tasks compared to participants in the CBM-I Control condition. Finally, we hypothesized that participants with high levels of OC symptoms in the Positive CBM-I training condition would demonstrate the greatest change in interpretation bias.

## Method

### Ethical approval

The study was approved by the Human Research Ethics Committee of the University of New South Wales (HREC; HC11505), Sydney.

### Participants

Selected community participants were recruited through the following advertisement placed on local mental health and general community websites: *Are you someone bothered by intrusive thoughts or images that you find difficult to control? Do you find yourself repeating certain behaviours that you find difficult to resist? Researchers at the University of New South Wales are seeking participants for a study investigating beliefs, imagery, and behaviour.* Potential participants who responded to this advertisement were provided a secure link to access the online informed consent and pre-screening questionnaires. It is routine practice in our labs to screen for exclusion criteria for study integrity and ethical reasons. Exclusion criteria included: severe depression (PHQ9 > 19) or suicidal ideation (PHQ9 item 9 > 1), endorsement of substance abuse, a reported history of psychosis, and age < 18 years. Eligible participants completed the battery of baseline questionnaires (DOCS, OBQ) online.

### Measures

#### ***Dimensional obsessive-compulsive scale***

(DOCS; [30]). The DOCS is a 20-item measure that assesses the four dimensions of OC symptoms most reliably found in structural research of OCD symptom (Contamination, Responsibility, Unacceptable Thoughts, and Symmetry). Items are rated on a 5-point scale with total scores ranging from 0 to 80. The DOCS items were constructed based on evidence that obsessions and compulsions occur on a continuum of severity and therefore are suitable for both non-clinical and clinical respondents [30]. The DOCS has been validated in clinical and non-clinical samples and demonstrates excellent psychometric properties [30]. Mean scores in the clinical OCD validation sample were 30.03 (*SD* = 15.49) and in the healthy student sample were 11.93 (*SD* = 9.87). These mean values were used in the current study to refer to high DOCS and low DOCS, respectively. The recommended clinical cut-off score in classifying OCD patients from nonclinical adults is 18 [30]. Cronbach’s alpha was .94 in the current sample.

#### ***The obsessive beliefs questionnaire-TRIP***

The OBQ-TRIP (20-item version; [31]) is a factor-analytically derived brief version of the original Obsessive Compulsive Cognitions Working Group (OCCWG) 44-item version [17]. Each of the 20 items designed to measure cognitions and beliefs central to OCD are rated on a 7-point Likert-type scale (1 = disagree very much to 7 = agree very much). The OBQ-TRIP-20 correlates well with the full OBQ-TRIP and demonstrates good internal consistency, Cronbach’s alpha = .77-.82 [17]. Cronbach’s alpha was .95 in the current sample.

#### ***Patient health questionnaire***

The PHQ9 [32] is a self-report questionnaire consistent with the DSM-IV diagnostic criteria for major depressive disorder. A four-point frequency scale (0 = not at all, 3 = nearly every day) is used to rate each of the nine items. Higher scores are indicative of more severe depression symptoms (0–9 = normal, 10–14 = mild, 15–19 = moderate, 20–23 = severe, and 24–27 = very severe). The PHQ-9 demonstrates good psychometric properties [32]. Cronbach’s alpha was .86 in the current sample.

#### ***OC bias index***

To obtain an index of interpretation bias both before and after CBM-I training, the bias measure of Clerkin and Teachman [28] was employed. Participants were first exposed to 10 scenarios with a missing letter in the final word of the sentence. Each scenario remained ambiguous in nature even after completion of the word fragment (e.g., '*You are driving to visit friends who live several hours away. Outside, it begins to rain and you are careful to drive the speed limit. You think about the importance of driving s_fely’*). In this example, the letter 'a’ would be required to complete the word stem of 'safely’. Following the filler task (below), participants were then randomly presented with 4 sentences and required to rate how similar each was to the meaning of the scenario they previously imagined themselves in (1 = *very different in meaning* to 4 *very similar in meaning*). Each sentence corresponded with four different interpretations, none of which was worded identically to the sentence in the paragraph they had previously imagined themselves in. OC-Positive scenarios were consistent with a response that challenges the core maladaptive belief (e.g., '*As you drive down the road, you think your chances of getting into an accident are low because you are being so cautious’*) whereas OC-Negative scenarios were those consistent with a response that reinforces the core maladaptive belief (e.g., '*As you drive down the road, you worry that you’ll accidentally crash your car even though you aren’t speeding’*). Foil scenarios were included to assess for a general interpretation bias. Foil Positive scenarios were positive, but unrelated to core OC maladaptive beliefs (e.g., '*As you drive down the road, you are looking forward to visiting your friend’*) and Foil Negative scenarios were negative, but also unrelated to OC beliefs (e.g., '*As you drive down the road, you are not looking forward to visiting your friend’*). Cronbach’s alpha for the indices ranged from .68 to .79.

#### ***The positive and negative affect schedule***

(PANAS; [33]). The 10 item negative affect score was used to asses for state negative affect at baseline and following the CBM-I training task. Cronbach’s alpha was .90 in the current sample.

#### ***Filler task***

Participants were asked to rate the pleasantness of 60 neutral images taken from the International Affective Picture System [34] and piloted in Grisham, Becker, Williams, Whitton, and Makkar (unpublished). The images were displayed using Powerpoint after completion of CBM-I training to increase the believability of the cover story (that the researchers were interested in imagery) and to minimize any potential mood effects of training condition.

#### ***Behavioural tasks***

Three behavioural tasks were designed for the current study to provide an objective index of the effect of CBM-I training on emotional vulnerability. The first task was based on a thought-action fusion induction [35] employed in a number of experimental studies [28,36-38] to index Importance of Thoughts/Control. Participants were first instructed to type the name of an important person who is currently in the participant’s life in the space provided on the computer screen. The subsequent screen embedded the loved-one’s name in the following sentence '*Now imagine that (loved-one’s name) has been in a car accident’*. This information was presented on the computer screen for 30 s and then participants were asked to rate on a scale of 0 = 'not at all’ to 100 = 'Extremely’ the level of distress associated with the target thought and the degree to which they were actively attempting to suppress the information. Participants were also given the option to delete the sentence from the computer screen. An as index of Perfectionism/Intolerance of Uncertainty participants were asked to write a summary of the study procedures to be provided to the next participant. Participants were given only 45 seconds to enter their text response (pilot testing demonstrated that at least 90 seconds was required to adequately complete this task). Participants were then asked if they were confident with the accuracy of information they had provided and given the option to add additional text to their response. As an index of Contamination/Estimation of Threat, participants were asked to use disinfectant wipes to clean the computer keyboard and mouse under the premise that the computer space was a shared lab facility and that it was a University health and safety regulation requirement. The researcher covertly recorded the amount of time each participant spent cleaning the keyboard and counted the number of disinfectant wipes used.

### ***Procedure***

Following completion of the baseline screening and questionnaires, eligible participants were then contacted by a research assistant to arrange the 1.5 hr laboratory session at which time they were allocated (based on the pre-defined randomisation sequence) to Condition and completed 1) the baseline mood measure (PANAS), 2) the baseline Scenario Bias Measure, 3) the filler task, 4) the CBM-I training task, 5) the filler task, 6) the post-training training mood measure (PANAS), the post-training Scenario Bias Measure, the OBQ-TRIP, and finally the behavioural tasks. All participants then underwent a funneled debriefing process to ascertain suspicion regarding any aspects of the study.

#### ***CBM-I training task***

The CBM-I training task was based on existing protocols [28,39] that have demonstrated efficacy in inducing interpretation biases by resolving the ambiguity of potentially threatening information in a positive manner. Based on the methodology of Clerkin and Teachman [28] participants were asked to read and imagine themselves in various scenarios that could potentiate a negative OC interpretation. Scenarios were based on the individual items of the OBQ-44 [17] and tapped the broad belief domains of Tolerance for Uncertainty, Threat Estimation, Control of Thoughts, Importance of Thoughts, Responsibility and Perfectionism. In the current study, all scenarios were first pilot tested by obtaining ratings from 11 clinical psychologists specializing in the treatment of anxiety disorders and OCD. Each scenario was rated in terms of how well it related to the specific concept loading on each of the six belief domains on a scale of 1 = extremely poorly – 5 = extremely well. Scenarios with poor ratings were removed and replaced with modified items resulting in a final dataset of 164 scenarios. An example requiring a participant to resolve the ambiguity of a scenario tapping Threat Estimation (OBQ item: Avoiding serious problems, for example, illness or accidents, requires constant effort on my parts) by selecting the missing letter to complete the sentence is: '*You are riding the bus home from work. The passenger beside you sneezes so you offer them a tissue. You think to yourself that offering a tissue was a behaviour that was k_nd/ r_sky’* (requiring the participant to enter the letter 'i’ to form the word '*kind*’ in the positive condition or to form the word '*risky*’ in the control condition). The scenario completion task was followed by a comprehension question to ensure the participant had processed the meaning of the sentence '*Are you pleased that you offered a stranger a tissue?’* (YES/NO). In the Positive condition every training scenario had a positive resolution and in the Control condition half the scenarios had a positive resolution while the remaining scenarios had a negative resolution. Therefore a specific learning contingency was established between the ambiguous start of the scenario and a positive resolution, whereas in the control condition no such contingency was established.

### Data analytic approach

Sample size calculations were informed by effect sizes (ESs) reported in the most comparable study design [28] of a moderate between-group ES of training on interpretive bias of Cohen’s *d* = .76. We therefore estimated each condition to require a minimum of 22 participants per group (alpha = .05; power = .80), but more were recruited to hedge against potential data loss. Significance testing of group differences regarding demographic data and pre-treatment measurements were conducted using independent samples *t*-tests, and χ^2^ where the variables consisted of nominal data. General linear model (GLM) analyses were conducted with Time as a repeated factor and Condition as a within-subject variable to evaluate the impact of CBM-I on the primary outcome variables. To evaluate the potential impact of OCD symptoms (DOCS scores) on the effect of CBM-I, separate marginal model analyses using the restricted maximum likelihood (REML) estimation method were used. REML models are appropriate for pre-post only designs [40]. Model fit was determined using Schwarz's Bayesian Criterion (BIC). Based on the validation study of the DOCS [41], low values of DOCS scores were based on the mean of a healthy student sample (*M* = 12), whereas high values were based on the mean of the clinical OCD sample (*M* = 30). Effect sizes were calculated within groups (Cohen’s *d*) using the pooled standard deviation and adjusted for the repeated measure correlation. Effect sizes for between-group comparisons were calculated using Hedges *g* using the pooled standard deviation.

## Results

After removal of one participant who reportedly was receiving treatment for OCD, the final sample included 41 females and 48 males with a mean age of 35.67 (*SD* = 16.66). Chi-Square (*χ*^*2*^*)* and independent samples *t*-tests were first conducted to confirm that no significant differences existed between participants in the positive and control training conditions at baseline. There were no differences in gender [*χ*^*2*^ (1) = .59, *p* > .05], age [*t*(87) = 0.55, *p* >.05], self-report of any current anxiety or depressive disorder [*χ*^*2*^ (1) = .004, *p* > .05], or OC symptoms and the baseline measures or, all *p*s > .05 (see Table 1 for means). Thirty-one percent of the sample scored above the recommended clinical cut-off of 18 on the DOCS.

**Table 1:** Means (standard deviations) across training group

**Measure**	**Positive condition**	**Control condition**
	**(n = 41)**	**(n = 37)**
	**Mean (SE)**	**Mean (SE)**
**DOCS Total**	12.97 (12.25)	15.93 (11.60)
**OBQ-TRIP**	65.65 (29.27)	62.89 (26.05)
**PHQ9**	3.51 (4.28)	3.91 (3.69)
**PANAS Negative (Baseline)**	1.47 (.56)	1.45 (.57)
**PANAS Negative (Post-training)**	1.26 (.37)	1.28 (.37)

*Note:* DOCS = Dimensional Obsessive Compulsive Scale; OBQ –TRIP = Obsessive Beliefs Questionnaire; PHQ9 = Patient Health Questionnaire – Depression; PANAS = Positive Negative Affective Schedule – Negativity Score.

Data were first evaluated in terms of CBM-I training accuracy scores to determine whether participants understood and adhered to the training condition. A target accuracy rate of at least 75% was required. Eleven participants (n = 2 Positive; n = 9 Control) did not obtain this level of training accuracy and therefore were excluded from analyses. Independent samples *t*-test revealed that mean training accuracy was very high and did not differ across the Positive (90%) and Control (89%) conditions, *p* > .05. To reduce the potential for response bias to influence the results, data were also analysed with respect to participant’s beliefs regarding the purpose of the study. Forty-one percent (n = 32) did not report any suspicion regarding aspects of the study or knowledge of the purpose of the training task. The remaining participants reported some level of suspiciousness regarding aspects of the study. Analyses were conducted including these participants as none were able to identify the true purpose of the study.

To evaluate the effect of training on the primary outcome measures univariate GLM analyses were conducted with Time as the repeated factor and Condition as the within-subject variable. For Target Bias scores, there was a main effect of Time qualified by a significant Time by Condition interaction, [*F* (1, 76) = 15.02, *p* < .001]. Planned comparisons (see Table 2) revealed that bias scores significantly increased in the Positive condition (reflecting a bias away from negatively valenced interpretations and towards positively valenced interpretations). The shift in bias scores in the Control condition was not significant. Training condition superiority over the Control condition was observed, [*t*(76) = 2.91, *p* < .01], corresponding to a medium effect. For Foil Bias scores, the main effect of Time and the Time by Condition interaction was not significant, although the interaction approached significance, [*F*(1, 76) =3.53, *p* = .06].

**Table 2:** Effect of CBM-I training on bias scores across condition

	**Baseline**	**Post-training**	**Within**	**Within**	**Within ES**	**Between**	**Between ES**
	**Mean (SD)**	**Mean (SD)**	***r***	***t *(df)**	**(95% CI)**	***t *(df)**	**(95% CI)**
**Target Bias**							
Positive	-1.82 (5.90)	5.09 (7.12)	.36	7.27 (40)***	1.28 (.84 – 1.71)		
Control	-1.08 (6.92)	.48 (6.82)	.82	1.56 (36)		2.91 (76)**	.66 (.20 – 1.12)
**Foil Bias**							
Positive	2.58 (4.58)	4.04 (5.00)	.59	2.68 (40)	.37 (-.06 – .80)		
Control	2.27 (2.73)	2.24 (4.07)	.86	.04 (36)		2.08 (87)	.38 (-.05 – .84)

*Note:* ***p < .001, **p < .01, *p < .05; Within Effect Size = Cohens *d*; Between Effect Size = Hedges *g*.

To confirm that state mood effects could not account for the training effect, PANAS Negativity scores were evaluated using repeated measures GLM. There was a main effect of Time [*F* (1, 75) = 15.16 *p* < .001], indicating a reduction in negative mood following training [*M* = 1.26 (*SD* = .37) and *M* = 1.28 (*SD* = .37) for the Positive and Control conditions, respectively – see Table 1 for baseline means], but importantly the Time by Condition interaction was not significant, *p* > .05.

### The impact of CBM-I on behavioural measures

For the Importance/Control of Thoughts task, independent samples *t*-tests revealed a significant difference in mean ratings of distress in the Positive (*M* = 3.85, *SD* = .97) relative to Control condition (*M* = 4.32, *SD* = .88) and urge to neutralize in the Positive (*M* = 3.37, *SD* = 1.00) relative to Control condition (*M* = 3.86, *SD* = 1.10), [*t*(75)s > 2.03, *p*s < .05], however, there was no difference in the number of participants who elected to erase the sentence, [*χ*^*2*^ (1) = .70, *p* > .05]. For the Perfectionism/Intolerance of Uncertainty task, there were no differences in endorsement of confidence or in the number of participants who elected to add additional information, [*χ*^*2*^ (1) = .01, *p* > .05]. For the Contamination/Estimation of Threat task, there were no differences in the amount of disinfectant wipes used in the Positive and Control condition (*M* =1.32, *SD* = .57 and *M* =1.38, *SD* = .55, respectively), or in the total amount of time (in seconds) participants spent cleaning the keyboard, (*M* = 57.64, *SD* = 29.83 and 55.09, *SD* = 27.61, respectively), *t*s < .5, *p*s > .05 ^a^.

### The impact of OCD symptom severity on CBM-I

**Figure 1:**
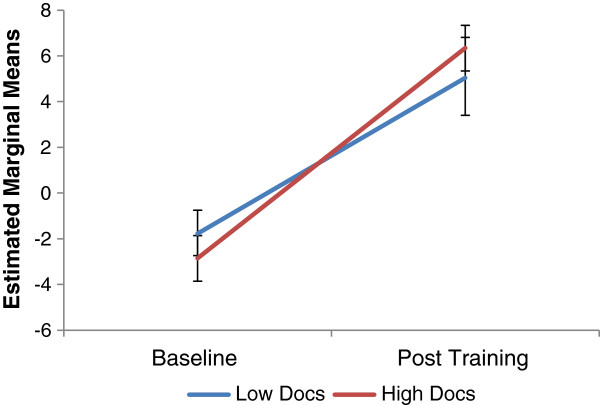
Estimated marginal means (standard errors) for Target Bias scores at the level of low and high DOCS in the positive condition.

To evaluate the influence of OCD symptom severity on training outcome, marginal model analyses were then conducted including baseline DOCS Total scores as a covariate and as an interaction term. Analyses were conducted separately for the Target Bias and Foil Bias scores. For each model, time was entered as a factor and the DOCS Total score and the time by DOCS Total score interaction term were entered as fixed covariates. Estimated marginal means and standard errors for the value of Target Bias and Foil Bias scores at the level of Low DOC scores (DOCS Total =12) and High DOCS scores (DOCS Total = 30) are reported in Table 3 and Figure 1. For Target Bias scores, the main effects of Time and DOCS were qualified by a significant Time × Condition × DOCS interaction, [*F* (2, 74) = 4.67, *p* < .05]. To dismantle the 3-way interaction, pairwise contrasts were conducted. Results are reported in Table 3 that demonstrate there was a significant increase towards a positive interpretation bias in the Positive group for target scenarios, irrespective of OC status. The significant interaction in the overall model was accounted for by the significant increase in Target Bias scores at the level of Low DOCS scores that was not observed at the level of High DOCS scores in the Control condition. For Foil Bias scores, there were no main effects or significant interactions, all *p*s > .05.

**Table 3:** Effect of CBM-I training on bias scores at the level of low and high OC symptoms

	**Low DOCS**	**Low DOCS**	**High DOCS**	**High DOCS**
	**Baseline**	**Post-training**	**Baseline**	**Post-training**
	**E Mean (SE)**	**E Mean (SE)**	**E Mean (SE)**	**E Mean (SE)**
**Positive training**				
Target Bias	-1.78 (.96)	5.04 (1.03)***	-2.86 (1.64)	6.34 (1.77)***
Foil Bias	2.72 (.57)	4.06 (.68)	2.17 (.98)	5.02 (1.17)
**Control training**				
Target Bias	-.30 (1.04)	1.42 (1.12)*	-5.13 (1.73)	-4.43 (1.85)
Foil Bias	2.58 (.58)	2.76 (.69)	2.28 (.87)	2.14 (1.03)

*Note:* E Mean = estimated marginal mean. DOCS = Dimensional Obsessive Compulsive Scale; Low DOCS = 12; High DOCS = 30. ***p < .001, *p < .05. Significant mean differences refer to baseline to post-training change in bias scores.

## Discussion

The current study aimed to extend initial research demonstrating the benefit of CBM-I training on negative interpretation biases associated with OC symptomatology. We first evaluated the impact of CBM-I on measures of interpretation bias. As predicted, community-recruited participants assigned to the Positive CBM-I training condition demonstrated a change in interpretation bias towards more positive and less negative OC-relevant interpretations compared to participants assigned to the Control CBM-I training condition. These results align with the preliminary findings of Clerkin and Teachman [28] who reported a significant effect of CBM-I training on OC interpretation biases in a student sample with high OC symptoms. Importantly, there was not a corresponding shift in interpretive bias to the foil scenarios that were unrelated to the core OC belief domains, suggesting specificity in the CBM-I training effect.

A further aim of the current study was to extend the findings of the effects of CBM-I training on self-report measures of bias to the behavioural domain. We utilized behavioural tasks designed to tap the core belief domains of Importance/Control of Thoughts, Perfectionism/Intolerance of Uncertainty, and Contamination/Estimation of Threat. Although self-report ratings of distress and urge to neutralize in response to the Importance/Control of Thoughts task were lower in the Positive CBM-I condition following training, differences were not observed between the conditions on the behavioural index associated with this task (deleting the accident target scenario). Additionally, no differential response was evident on any of the behavioural task measures designed to tap Perfectionism/Intolerance of Uncertainty or Contamination/Estimation of Threat. Several explanations may account for these null findings. Firstly, the behavioural tasks needed to be developed within the constraints of the research session and presented in a manner to maintain participant believability. Therefore the nature of the tasks may have prevented sufficient levels of anxiety or OC-specific cognitions to become activated. Secondly, measurements were in the form of either a dichotomous outcome variable or a response with limited variability, and therefore may have lacked sensitivity to detect differences between the conditions. Further, research demonstrates that there is often a lack of concordance between self-report and behavioural measures. It has been suggested that behavioural measures may capture specific aspects of behaviour that are functionally distinct from the broader range of thoughts, feelings, and self-acknowledged behaviours indexed by self-report measures [41].

Finally, in order to demonstrate the specificity of the observed training effect on participants most likely to be vulnerable to maladaptive OC beliefs, we evaluated the impact of high vs. low OC symptoms on the effects of CBM-I. Contrary to prediction, results demonstrated that participants in the Positive CBM-I condition evidenced a significant shift towards more positive interpretations of target scenarios, irrespective of OC status. Although these results do not support a differential response to CBM-I based on OC symptom severity, the overall results do support the specific benefit of CBM-I training on information that is relevant to individuals with high levels of OC symptoms. Importantly, the effects of CBM-I did not reflect a general positivity bias as participants did not evidence a shift in interpretation bias in response to the foil scenarios. Interestingly, results indicated a positive shift in interpretative bias in Control condition participants who scored in the healthy range on the DOCS. It is possible that the degree of exposure to scenarios with a positive resolution (in 50% of the cases) was sufficient to shift interpretations of information that is unlikely to be regularly cognitively activated by individuals with low levels of OC symptoms (e.g., *As you think about this bizarre thought, you decide it is significant and says something important about your personal values).*

These results of the current study must be considered in light of a number of limitations. The CBM-I training session, although consisting of 164 different OC-relevant scenarios, was delivered in a single session. It is possible that a more protracted session, or a series of multiple sessions delivered over several days, would be required to impact upon behavioural responding. Future studies would benefit from manipulations of the number of CBM-I sessions required for optimal effects. The sample was comprised of community members with varying levels of OC symptoms, therefore the results may not generalize to clinical samples of individuals with OCD. Our research group is currently planning a series of studies with clinical samples to ensure the effects of CBM-I do generalize, and further, to assess whether the effects can impact upon behavioural approach tasks that are more representative of the concerns experienced by clinical populations.

## Conclusions

Future research should examine the utility of integrating CBM-I paradigms with existing evidence-based interventions. It has been noted by leading authorities in the field [42] that CBM procedures lend themselves to being delivered remotely and are cost-effective, making them an ideal candidate for inclusion in internet-delivered cognitive behavioural therapy (iCBT). Evidence is emerging that the two types of interventions can be effectively combined in the treatment of other clinical disorders [21], therefore future research should aim to establish the combined efficacy in the treatment of OCD. As only approximately 42% of individuals with OCD receive evidence-based treatments [43], the need for treatment innovation and alternative service delivery methods is clear.

## Endnote

^a^Data for 16 participants was excluded on this task due to either participant suspicion or administrator error.

## Competing interests

The authors declare that they have no competing interests.

## Authors’ contributions

AW conceived of the study. AW and JG coordinated the study design. AW performed the statistical analysis. AW and JG wrote the manuscript and approved the final manuscript. Both authors read and approved the final manuscript.

## Pre-publication history

The pre-publication history for this paper can be accessed here:

http://www.biomedcentral.com/1471-244X/13/256/prepub
